# My Experience with Jequirity in the Treatment of Granular Eyelids

**Published:** 1884-02

**Authors:** F. C. Hotz

**Affiliations:** Chicago


### ARTICLE II.

### MY EXPERIENCE WITH JEQUIRITY IN THE TREATMENT OF GRANULAR EYELIDS. By F. C. HOTZ, M.D., Chicago. Read before the Chicago Medical Society, December 3, 1883.

A little over a year ago Dr. Wecker, of Paris, to whom ophthalmology is indebted for the introduction of several valuable and useful remedies, announced the discovery of a remedy which seemed to possess a healing power surpassing anything heretofore used in the treatment of granular conjunctivitis. He introduced the remedy under the odd term "jequirity," which is only another name for what in this country is popularly known as sea beans, or "crab eyes," though these red, egg-shaped peas which you see here, grow neither in the sea nor in crabs, but are the seeds of a South American plant, *abrus precatorius* (also known as "wild liquorice," and by half a dozen other names).

Among the natives of Brazil these peas had long enjoyed a great popularity as a good remedy for inflamed eyes, and the discovery of their important medicinal properties by Dr. Wecker was the result of a mere accident. A Brazilian gentleman, who at different times had been a patient of Dr. Wecker's but never been fully relieved of the trachoma of his eyelids, was induced to try the popular home remedy. The quick and complete relief he experienced after its use of course gave him a very high opinion of the remedy, and being of a sympathetic nature probably he pitied the multitude of sufferers whom he saw wandering in the streets of the European capitals from oculist to oculist and from one hospital to another seeking, but never finding a permanent relief for the granulated eyelids. So this gentleman sent a package of sea beans to Dr. Wecker with a full description of the manner the natives use them, and with an urgent request to try them, giving his own case as a reference for their efficiency..

Dr. Wecker complied with this request, following at first strictly the directions of the natives, who prepared a weak infusion (0.3 per cent.) with which they bathe the eyes three times per day for three consecutive days ; then he proceeded to test the effect of infusions of various strength, increasing it to 2, 3 and 5 per cent.* His experiments soon convinced the doctor of these facts :

1. That jequirity has a most remarkable effect upon the conjunctiva, producing a peculiar, violent, acute inflammation.

2. That the severity of this inflammation can be graded by the strength of the infusion and the number of applications.

3. That this jequirity conjunctivitis involves no danger to the eye, and

4. That it cures the granulations and clears the vascular corneæ quickly and thoroughly.

* His prescription for the 5 per cent. infusion is as follows: Take 10 grams of the skinned seeds, pulverize (not only crush) them well; add 50 grams of cold water, leave it stand twenty-four hours, then filter before using. I have recently found that the infusion is just as effective if the seeds have been macerated only ten minutes.

That such statements, coming from such a source, should attract a good deal of attention among oculists, was very natural, considering the present state of our therapy for granular eyelids. With the most approved methods the treatment is slow, tedious, and uncertain of its success. No wonder that Dr. Wecker's accounts of his remarkably quick cures by a few applications of jequirity were read by many with a great deal of skepticism. And I confess I belonged to the skeptical class until last summer, when Dr. Gruening, of New York, showed me in a number of of cases so marvellous results, and spoke so warmly for the remedy that I became greatly interested, and resolved to test it. The material for these tests was found in abundance at the Eye and Ear Infirmary; for, being a State institution, it receives the poor patients from all parts of the State of Illinois, and therefore among its inmates there is always a great number of patients afflicted with granular ophthalmia in its various forms and phases, from the fresh acute attack to the old chronic form, with atrophy of the conjunctiva, entropium, ulceration and vascularization of the cornea.* Assured that the jequirity treatment does not injure the eye, I thought the experiment was a legitimate one and, worth the trial, inasmuch as a quick recovery would be a great blessing to those poor patients and a financial gain for the State.

I began my experiments in July. At first a 2-per-cent. infusion was used twice or three times daily for two or three days, according to the severity of the reaction produced. Later on (after I had read Dr. Wecker's paper in the July number of the *Klin. Monatsblötter*, where he recommended the 5-per-cent. infusion) the strength of the infusion was increased to 5 per cent., but the number of applications reduced to one per day, and with this stronger lotion two applications (and in a few cases even one) were sufficient to produce the typical jequirity inflammation. The infusion was applied upon the conjunctiva of the everted eyelids by means of a camel's-hair brush.

The application itself was always entirely painless, and not till six or eight hours later did the patient begin to feel the action of

* For instance, among the 393 house patients of the Eye department admitted during the year ending September 30, 1883, there were 115 cases of granular lids with and without pannus.

the medicine, by pain and irritation of his eyes. After twenty-four hours there always was already more or less œdema of the eyelids, much redness and swelling of the conjunctiva, discharge of a muco-purulent character, and the patient complained of considerable pain and great tenderness of the eye. Under these circumstances the everting of the upper lids for making the second application of the medicine was very difficult and painful, but the medicine itself, also then, caused no pain. The night following the second application was the worst time for the patient. He suffered agonies of pain and walked the floor unless given a large dose ot an opiate, and in sensitive persons the violent local inflammation reacted upon the whole system, causing fever and general malaise. The swelling of the eyelids attained such enormous dimensions, that eyelashes and lid-borders were completely invisible, and that it became exceedingly difficult to open the eye. The tarsal and retro-tarsal conjunctiva was covered with a thick, white coat of a croupous exudation; the ocular conjunctiva was very red and swollen, so that it projected far over the margin of the cornea. The cornea always showed remarkable changes: where it had been vascular and hazy before the treatment, it was more so now, and where it had been transparent before, it became dull, lusterless, and gray.

Those symptoms of the fully developed jequirity ophthalmia are frightful, indeed, and may fill the mind with anxiety for the safety of the eye; and time and again have physicians, when looking at such an eye, betrayed, either by the expression of their faces or by remarks, that they entertained serious doubts regarding the harmlessness of the treatment, any assurances to this effect notwithstanding. But if this condition of the eye can alarm the physician, how must it frighten the patient if he was not prepared for it. I have, therefore, told every patient, before I applied the remedy, that it would inflame his eyes very badly, and that he would suffer considerably for one day; but at the same time I gave him the firm assurance that there was not the least danger, that his eye would safely get over the inflammation in a few days, and then quickly get well. I made it a rule to give this information and explanation in every case, and I have good reasons for being satisfied with having done so, and I would recommend this rule to every one who wishes to use jequirity. It has a wonderful, strengthening effect upon the faith and moral courage of the patient, when he hears his physician speak of the direct effect and the ultimate result of the proposed treatment in such precise and positive terms, and his faith in your medical experience will become unbounded when he finds afterwards that everything passed off exactly as you predicted.

When the typical effect of the jequirity, the croupous-blennorhœal ophthalmia, was obtained, the remedy was discontinued. The patient remained in his room for two or three days, bathing his eyes frequently with water to remove the discharge. When the pain was unusually severe and the swelling excessive, ice-cold compresses were applied during the day, and opiates given at night. Pain and swelling, however, subsided always very quickly, the pain seldom lasting more than 24 hours; and two days after the last treatment the œdema of the eyelids had usually pretty well disappeared. The croupous exudation was then thrown off, and within one week the eye had recovered from the immediate effect of the jequirity treatment.

During this time already, but more so in the second and third weeks, the wonderful effect of jequirity upon the granulations of the conjunctiva and the pannus of the cornea became manifest. In the successful cases, at the end of the third week, the conjunctiva was as nearly normal as this was possible under the circumstances; or, in other words, the few treatments thoroughly eradicated the granulations, and removed all thickening and roughness of the conjunctiva; but, of course, they could not remove atrophic patches and cicatrices previously produced in the conjunctiva by the disease.

The action of jequirity seemed equally effective whether enlarged papillœ or lymph follicles constituted the predominant feature of the granular disease. I paid especial attention to this point, but failed to discover any marked difference; the most cases of the papillary type were cursed by the first jequiritization, a few required a second course of the same treatment; but the same can be said of the follicular and of the mixed forms. Only one case (Grimm) with well developed granulations (papillary form) was not benefitted by the treatment, though it produced a very severe and typical jequirity ophthalmia. But I am inclined to attribute this failure to the fact that at the time of jequiritization the eyes were in the state of acute inflammation, a very severe acute relapse of inflammation just having attacked the lids and the cornea. We know that acute granular conjunctivitis and acute keratitis (pannus) do not tolerate the application of stimulants or irritants. This has been the universal experience with all the various modes of treatment heretofore, and I believe jequirity will prove no exception to this rule. In the case referred to it affirmed the rule; for it decidedly intensified and prolonged the acute inflammation. After this experience I have always subdued every symptoms of acute inflammation by the frequent use of ice water compresses before the jequirity was applied, and I have not had another failure since.

There is another class of cases which received no benefit from jequirity. But they really do not belong to the class of granular eyelids; they are a peculiar form of catarrhal conjunctivitis, its peculiar feature being this: That the inflammation affects chiefly the retro-tarsal portion of the conjunctiva. This portion is infiltrated, swollen, succulent and sometimes thickly studded with lymph-follicles, while the tarsal conjunctiva is smooth and thin showing perhaps only a few isolated small follicles. I treated three cases of this kind with jequirity, and none were materially benefitted by the treatment.

Now, what was the effect of jequiritization on the cornea? In the first place, I will say, that my experience fully agrees with Dr. Wecker's in this important fact, to-wit: That in no case the cornea received the least injury or damage from the effect of jequirity. It was used upon eyes with normal cornæ as well as upon eyes with ulcerations, pannus, leucoma of the cornea; but not in one single instance have I noticed any ill effect. During the acme of jequirity ophthalmia, it is true, the cornea became gray and dull, but with the subsidence of the inflammation it always recovered its normal transparency and bright lustre. Corneal ulcers neither got any worse nor was their repair materially promoted by jequirity.

Non-vascular opacities of the cornea (leucoma) were not affected at all. But most remarkable was the effect upon the vascular-opacities (pannus) of the cornea. I saw the densest pannus which had reduced the sight to the bare perception of light (just about as much as you can see through your closed eyelids) clear away within two weeks so much that the patient could easily walk alone and decipher large type. And this result was obtained in cases which, for many months past, had shown no sign of improvement, though no remedy was left untried save the operation of peritomy and the inoculation of blennorrhœa. And, indeed, it seemed to me that these cases of inveterate pannus, which resisted all other medicines, yielded the best results; here the jequirity accomplished wonderful changes and showed its virtues in the most brilliant light. The first favorable sign, I always noticed, was the brighter luster of the cornea; then the blood vessels on its surface became thinner and receded gradually from the center to the margin, while the epithelial layers of the cornea became visibly clearer.

So successful was the effect of the jequirity upon the cornea that only in three eyes out of thirty-six it failed to remove the pannus. In one eye the conjunctiva was so atrophied that the 5-per-cent. lotion of jequirity could not produce any inflammation, though it was used every day during a whole week. The other two eyes were those of that patient (Grimm) mentioned above, which were treated while in the state of acute inflammation.

Summing up my experience gathered from the study of 65 eyes (50 in the Eye and Ear Infirmary, 15 in private practice) which I treated with jequirity from July to November, I can express it in the following propositions:

1, Jequirity is the best known remedy for the chronic granular conjunctivitis.

2. It is the most effective remedy for the clearing of trachomatous pannus, and in inveterate forms of pannus it is preferable to peritomy, as well as to the inoculation of blennorrhœal virus, because it does its work quicker than the operation, and safer than the inoculation.

3. It has no injurious effect upon the eye, and can be used with perfect safety, even when the cornea is ulcerated.

4. But it should not be used while the cornea and conjunctiva are acutely inflamed.

5. It does not benefit those cases of chronic conjunctivitis in which the symptoms of catarrh (increased secretion, succulence of the retro-tarsal folds, etc.) predominate over those of trachoma (enlarged papillæ, and lymph follicles, plastic infiltration of tarsal conjunctiva).

6. The most violent attacks of jequirity ophthalmia accomplish the speediest cures of granulated eyelids and the quickest clearing up of the vascular cornea.

